# Genome-Wide Identification and Expression Analysis of m6A Writers, Erasers, and Readers in Litchi (*Litchi chinensis* Sonn.)

**DOI:** 10.3390/genes13122284

**Published:** 2022-12-04

**Authors:** Liwen Tang, Jiali Xue, Xingyu Ren, Yue Zhang, Liqing Du, Feng Ding, Kaibing Zhou, Wuqiang Ma

**Affiliations:** 1 Sanya Nanfan Research Institute of Hainan University, Sanya 572025, China; 2 Key Laboratory for Quality Regulation of Tropical Horticultural Crops of Hainan Province, Horticulture College, School of Horticulture, Haikou 570228, China; 3 Key Laboratory of Tropical Fruit Biology, Ministry of Agriculture & Rural Affairs, South Subtropical Crops Research Institute of Chinese Academy of Tropical Agricultural Sciences, Zhanjiang 524091, China; 4 Guangxi Crop Genetic Improvement and Biotechnology Key Laboratory, Guangxi Academy of Agricultural Sciences, Nanning 530007, China

**Keywords:** litchi, m6A modification, writers, erasers, readers, expression analysis, fruit coloration, fruit abscission

## Abstract

N6-methyladenosine (m6A) RNA modification is the most prevalent type of RNA methylation and plays a pivotal role in the development of plants. However, knowledge of the m6A modification in litchi remains limited. In this study, a complete analysis of m6A writers, erasers, and readers in litchi was performed and 31 litchi m6A regulatory genes were identified in total, including 7 m6A writers, 12 m6A erases, and 12 readers. Phylogeny analysis showed that all three of the kinds of litchi m6A regulatory proteins could be divided into three groups; domains and motifs exhibited similar patterns in the same group. MiRNA target site prediction showed that 77 miRNA target sites were located in 25 (80.6%) litchi m6A regulatory genes. Cis-elements analysis exhibited that litchi m6A regulatory genes were mainly responsive to light and plant hormones, followed by environmental stress and plant development. Expression analysis revealed litchi m6A regulatory genes might play an important role during the peel coloration and fruit abscission of litchi. This study provided valuable and expectable information of litchi m6A regulatory genes and their potential epigenetic regulation mechanism in litchi.

## 1. Introduction

*N^6^*-methyladenosine (m6A) RNA methylation occurs at the sixth N atom of adenine and plays an important role in the post transcriptional regulation of genes in the eukaryote world [1]. m6A modification is the best-identified and most prevalent type of RNA methylation, accounting for up to 80% of RNA methylation modifications in eukaryotes and 50% of methylated nucleotides in message RNA (mRNA) [2]. The pathway of m6A modification is conserved, dynamic, and reversible, and it is mainly regulated by three categorical proteins, namely writers (methyltransferases), erasers (demethylases), and readers (biding proteins), respectively [1]. In plants, writers primarily include MTA, MTB, MTC, VIR, HAKA, and FIP proteins, functioning to add m6A modification to the following conserved motif sequences: RR[A]CH (R = G/A, H = A/C/U, [A] = m6A), URU[A]Y(R = G/A; Y = C/U), and their similar sequences [3,4]. Erasers containing ALKBH9B and ALKBH10B proteins are responsible for removing the m6A modification [5,6]. Readers embodying ETC, CPSF30, and HNRNP proteins contribute to the recognition of the site of m6A modification to activate the downstream regulatory pathway [7]. 

m6A modification widely exists in eukaryotes, and research in this field mainly focuses on mammals and fungi nowadays [8,9]; there are far fewer reports of m6A modification in plants, which just shows in the common model plants, such as wheat (*Triticum turgidum* L.) [10], arabidopsis (*Arabidopsis thaliana*) [11], rice (*Oryza sativa* L.) [4], tomato (*Lycopersicon esculentum* Miller.) [12,13], and maize (*Zea mays* L.) [14]. The function of m6A modification depends on the expression of m6A regulatory genes. The core set of m6A writer proteins in arabidopsis, including MTA, MTB, FIP37, VIRILIZER, and HAKAI, played a role in developmental decisions during pattern formation; low levels of these proteins induced a decline in the relative m6A levels and shared pleiotropic phenotypes, which included aberrant vascular formation in the root [15]. OsFIP37 could be used by an RNA-binding protein (OsFAP1) to mediate m6A modification on an auxin biosynthesis gene (OsYUCCA3) to keep stabilized, which was essential for meiotic division and subsequent pollen development in rice [16]. The depletion of ALKBH10B, an eraser protein in arabidopsis, could delay flowering and repress vegetative growth [6]. SlALKBH2 could bind the transcript of *SlDML2* (a DNA demethylase gene required for tomato fruit ripening) to modulate its stability via m6A demethylation; the mutation of SlALKBH2 reduced the abundance of *SlDML2* and delayed fruit ripening [12]. The injection of exogenous 3-deazaneplanocin A (DA; m6A writer inhibitor) or meclofenamic acid (MA; m6A eraser inhibitor) into tomato fruits could alter the whole m6A level, suppressing fruit expansion while, simultaneously, accelerating or delaying fruit ripening, respectively [13]. Transcriptome-wide m6A analysis revealed that two writers (*MTB* and *TRM4A*), one reader (*CPSF30*), and one eraser (*ALKBH9A*) exhibited differential expressions in the roots of rice cultivar *Nipponbare* under salt conditions [17]. ECT1 and ECT2 readers in arabidopsis could interact with the stress response protein calcineurin B-like-interacting protein kinase 1 (CIPK1) specifically and played a vital role in the transmission of calcium signal to the plant nucleus under various external stimuli [18]. All the studies mentioned above suggest that the m6A modification plays a vital role in plant growth, development, and stress response [1]. 

Litchi (*Litchi chinensis* Sonn.), a kind of evergreen tropical and subtropical fruit tree belongs to the sapindraceae family, notable for its bright appearance and high nutritional and commercial value. However, a series of problems still exist, such as ‘stay green’(when fruit has reached its best edible period, the peel cannot maintain coloration or it has uneven pigmenting) in some of the main varieties, such as ‘Feizixiao’ [19], and the phenomenon of serious fruit abscission (including physiological fruit drop and fruit drop induced by external environments) in almost all of the litchi varieties [20]. The m6A modification has been reported to be closely related to important biological functions in the plants [1]. However, knowledge of the m6A-regulated pathway in litchi remains limited. In this study, genome-wide identification of litchi m6A-regulated pathway genes (writers, erasers, and readers) was executed with the help of recently released genome data [21], and their physicochemical properties, such as phylogenetic relationship, gene and protein structure, miRNA target sites, chromosomal arrangement, *cis*-regulatory elements, and expression, were comprehensively analyzed. This work aims to supply a solid foundation for the functional exploration of m6A-regulated pathway genes in litchi and other plants.

## 2. Materials and Methods

### 2.1. Identification of m6A Regulatory Genes in Litchi

The known protein sequences of m6A writers, erasers, and readers in *Arabidopsis thaliana* were downloaded from TAIR database (https://www.arabidopsis.org/, accessed on 3 March 2021). Homology search was conducted between arabidopsis (*Arabidopsis thaliana*) m6A regulatory proteins and all proteins of litchi at chromosome level [21] using TBtools software [22]. Conserved and typical domains of m6A writers, erasers, and readers, such as the MT-A70 domain in MTA, MTB, and MTB proteins; WTAP domain in FIP37 proteins; YTH domain in ECT and CFPF30 proteins; VIR_N domain in virilizer proteins; and 20G-Fell_Oxy_2 domain in ALKBM proteins, were confirmed depending on the Pfam database (http://pfam.xfam.org/, accessed on 9 May 2021) and Hmmer2.3 database (http://hmmer.janelia.org/, accessed on 9 May 2021). The sequences that did not contain the above domains would be deleted, and the rest were considered as litchi m6A writers, erasers, and readers.

### 2.2. Physicochemical Properties Analysis

The ExPASY website (http://web.expasy.org/protparam/, accessed on 22 April 2021) was used to analyze the physicochemical properties of litchi m6A proteins, such as isoelectric point (PI), molecular weight (MW), instability index, aliphatic index, and grand average of hydropathicity (GRAVY). The BUSCA website (http://busca.biocomp.unibo.it/, accessed on 22 July 2022) and Plant-mPLoc (http://www.csbio.sjtu.edu.cn/bioinf/plant-multi/, accessed on 22 July 2022) were adopted to predict their subcellular localization. The MBC website (http://cello.life.nctu.edu.tw, accessed on 22 July 2022) was applied to predict signal peptide and transmembrane structure.

### 2.3. Phylogenetic Analysis

The protein sequences of m6A writers, erasers, and readers of arabidopsis and litchi were used to complete phylogenetic analysis. Clustal X2 and MEGA 6 software were adopted to construct the phylogenetic tree, which depended on neighbor joining (Nj) algorithm with 1000 bootstrap replicates, and other parameters were default settings. All litchi m6A regulatory genes were renamed according to their homologs of the arabidopsis m6A regulatory genes. 

### 2.4. Gene Structure, Conserved Domain, and Conserved Motif Analysis

The gene structure coordinated information of litchi m6A regulatory genes obtained by the gff file of litchi genome [21]. NCBI cd-search website (https://www.ncbi.nlm.nih.gov/Structure/bwrpsb/bwrpsb.cgi, accessed on 23 July 2022) and SMART database (http://smart.embl-heidelberg.de/, accessed on 23 July 2022) were used to identify the conserved domains. The MEME tools (http://meme-suite.org/tools/meme, accessed on 23 July 2022) were adopted to complete the motif analysis. Lastly, the gene structure, conserved domain, and conserved motif of litchi m6A regulatory genes were displayed by the TBtools software [22].

### 2.5. Prediction of MiRNA Target Site

The psRNATarget website (https://www.zhaolab.org/psRNATarget/analysis?function=3, accessed on 14 August 2022) was employed to perform the miRNA target site prediction, combined with the litchi miRNAs sequences obtained from the previous pieces of work [23], where the litchi m6A regulatory genes sequences used default parameter settings. The miRNA target site distribution was shown on the CDS sequence of each litchi m6A regulatory gene by TBtools software [22].

### 2.6. Chromosomal Arrangement and Gene Ontology (GO) Enrichment Analysis 

The chromosomal location information of litchi m6A regulatory genes were obtained from the genome annotation files of litchi [21] and exhibited by the TBtools software [22]. As for GO analysis, firstly, all litchi genes were blasted to the uniprot_sprot.fasta file downloaded from the Swissprot database (https://www.uniprot.org/, accessed on 12 June 2022), then GO annotation and enrichment analysis were completed by the TBtools software [22]. 

### 2.7. The 3D Protein Structure Analysis 

GOR IV (https://npsa-prabi.ibcp.fr/cgi-bin/npsa_automat.pl?page=/NPSA/npsa_gor4.html, accessed on 26 July 2022) was used to predict protein secondary structure, and the SWISS-MODEL online tools (https://swissmodel.expasy.org/, accessed on 26 July 2022) were adopted to predict the 3D protein structure.

### 2.8. Cis-Regulatory Elements Analysis

The promoter region sequences of litchi m6A regulatory genes (2000 bp upstream sequences from the translation initiation codon ‘ATG’) were grabbed. The Plant Care database (http://bioinformatics.psb.ugent.be/webtools/plantcare/html/, accessed on 12 June 2022) was adopted to do the *cis*-regulatory elements analysis. The TBtools software [22] was used to exhibit the distribution patterns of *cis*-regulatory elements.

### 2.9. Expression Analysis of Litchi m6A Regulatory Genes RNA-Seq Data

Four sets of RNA-seq data used for the expression analysis of litchi m6A regulatory genes are as follows: (1) The peel samples of two development fruit stages (complete green stage (the peel completely wraps the pulp) and the best edible stage) of ‘Feizixiao’ variety, which was treated by exogenous N-(2-Chloro-4-pyridyl)-N’-phenylurea (CPPU, which can inhibit the fruit coloration during the period of mature) (home data, not published yet). (2) The peel samples of three stages (green, yellow, and red peel stage) of ‘Nuomici’ variety (https://www.ncbi.nlm.nih.gov/sra/?term=SRP047115, accessed on 20 June 2022) [24]. (3) The fruitlet samples of ‘Wuye’ variety at 2 d, 4 d, and 7 d after fruitlet abscission induced by girdling plus defoliation treatment (GPD) (https://www.ncbi.nlm.nih.gov/sra/?term=SRA234477, accessed on 20 June 2022) [25]. (4) The abscission zone samples of ‘Feizixiao’ variety at 0 d, 1 d, 2 d, and 3 d after fruitlet abscission caused by exogenous ethephon (ETH) (https://www.ncbi.nlm.nih.gov/sra/?term=SRP173341, accessed on 20 June 2022) [26]. Detailed information of materials used in this study were listed in Table 1. The Salmon software was used to calculate the count value of litchi m6A regulatory genes [27]. Differential expression analysis was conducted by the edgeR tools on the OmicShare Cloud platform of Gene denovo Biotechnology Co., Ltd. (Guangzhou, China) (https://www.omicshare.com/tools/, accessed on 20 June 2022), with the following parameters: log2 (fold change) ≥ 1, *p* value < 0.05, and FDR < 0.05. Heatmaps were drawn by the TBtools software [22] with the log2 value of CPM values obtained from the results of edgeR tools. 

### 2.10. Expression Analysis of Litchi m6A Regulatory Genes Identified by Quantitative qRT-PCR

To further investigate the expression patterns of litchi m6A regulatory genes, four sets of litchi samples corresponding the above RNA-Seq data were gathered for the quantitative qRT-PCR analysis (qRT-PCR). Detailed material information could be seen in Table 1. The RNA Kit RNAiso Plus (#9108) and Fruit-mate (#9192) from Takara Biomedical Technology (Beijing) Co., Ltd., Beijing, China were used to extract the total RNA. The NanoPhotometer spectrophotometer (#Nano-600) from Jinpeng Analytical Instrument Co., Ltd. Shanghai, China) was adopted to check the quality and concentration of total RNA. The following kits: HiScript III 1st Strand cDNA Synthesis Kit (R312-02) and ChamQ Universal SYBR qRT-PCR Master Mix (Q711) from Vazyme Biotech Co., Ltd., Nanjing, China were employed to do reverse transcription and qRT-PCR separately. Two reference genes (*EF-1α* and *GAPDH*) [28] were selected and the 2^−ΔΔCt^ calculation method was conducted in this study. All the samples held three technical repetitions and difference analysis was accomplished by t-test in the SAS software. Primers used in this study were listed in Table S1.

## 3. Results

### 3.1. Identification of Litchi m6A Regulatory Genes

After homology search, a total of 31 litchi m6A regulatory genes were ultimately ascertained (Table 2), including 7 m6A writers (4 *LcFIP37s*, 2 *LcMTAs* (*LcMTB* and *LcMTC* were lost), and 1 *LcVirlizer*), 12 m6A erases (12 *LcALKBHs*), and 12 readers (8 *LcECTs*, 2 *LcHNRNPs,* and 2 *LcCPSF30s*). Physicochemical properties analysis showed that the length of amino acid varied from 104 to 2200 aa of writers, 236 to 1676 aa of erases, and 350 to 1514 aa of readers, respectively. The average molecular weight (MW) of these three kinds of litchi m6A regulatory proteins were 76.44, 57.22, and 70.43 kDa, respectively, and the average isoelectric point (PI) ranged from 4.75 to 9.51. The instability index of LcECT1&3, LcECT2&4, LcECT6&7, LcECT8, and LcHNRNP_1 were less than 40, revealing that they were stable proteins. Aliphatic index ranged from 39.81 to 99.33. The grand average of hydropathicity (GRAVY) analysis showed that litchi m6A regulatory proteins were hydrophilic protein. Subcellular localization prediction displayed that 24 litchi m6A regulatory proteins (77.4%) were located in the nucleus, 3 (LcALKBH8A, LcALKBH10A, and LcECT6&7) (9.7%) were located in chloroplast, 2 (LcALKBH1A and LcALKBH9B) (6.5%) were located in cytoplasm, 1 (LcALKBH2) (3.2%) was located in plasma membrane, and 1 (LcECT9) (3.2%) was located in extracellular space (Table 2).

### 3.2. Phylogenetic Relationship Analysis

**Figure 1:**
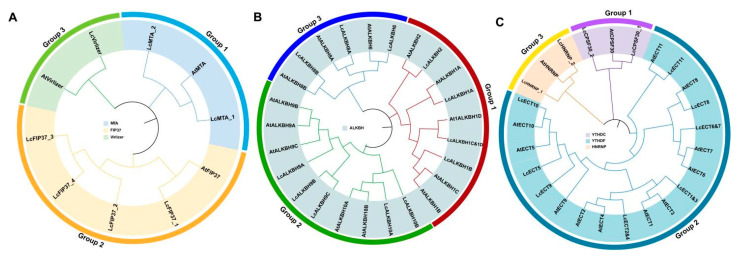
Phylogenetic trees of m6A regulatory genes of litchi and arabidopsis. (**A**) Writers; (**B**) Erasers; (**C**) Readers. The groups of m6A regulatory genes were shown in different colors.

To better understand the evolutionary relationships and classification of litchi m6A regulatory genes, the protein sequences of m6A writers, erasers, and readers of litchi and arabidopsis were adopted to construct the phylogenetic tree. The results exhibited that all three kinds of proteins could be divided into three groups (Figure 1). The writers in litchi included MTA, FIP37, and VIRLIZER families, but the MTB and MTC families were lost (Figure 1A). The erasers in litchi just embodied the ALKBH family (Figure 1B). The readers in litchi contained YTHDC, YTHDF, and HNRNP families (Figure 1C).

### 3.3. Structural Features Analysis

**Figure 2:**
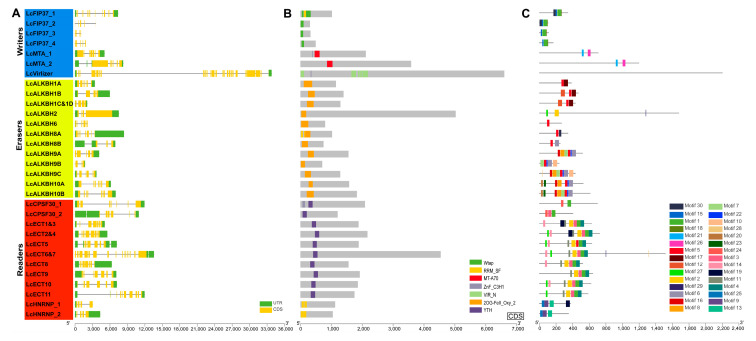
Gene structure, conserved domain, and motif of litchi m6A regulatory genes. (**A**) The distribution of gene structure. (**B**) The distribution of conserved domain. (**C**) The distribution of conserved motif.

Gene structure analysis showed that the numbers of exon of litchi m6A writers, erasers, and readers ranged from 5 to 28, 4 to 7, and 6 to 18, respectively. The exon–intron pattern of litchi m6A writers and readers exhibited much more variability than the erasers. Among these litchi m6A regulatory genes, *LcVirlizer* contained 28 exons and was the largest number, following were the *LcECT6&7* and *LcFIP37_1*, which possessed 18 and 14 exons, respectively. *LcALKBH1B*, *LcALKBH1C&1D*, *LcALKBH2,* and *LcALKBH9B*, which belonged to the erasers family, only owned four exons simultaneously (Figure 2A). Conserved domain analysis revealed that the typical WTAP, MT-A70, VIR_N, 20G-Fell_Oxy_2, YTH, and RRM_SF domains could be found in the LcFIP3, LcMTA, LcVIRLIZER, LcALKBM, LcECT, LcHNRNP, and LcCFPF30 family separately (Figure 2B and Table S2). The LcFIP37_2 and LcALKBH8A had one and two extra RRM_SF domains, respectively. LcMTA_1 and LcVirlizer had one additional ZnF_C3H1 domain separately. LcCFPF30_1 had three added ZnF_C3H1 domains, respectively (Figure 2B and Table S2). Motif analysis showed that, similar to conserved domain, these genes belonged to the same family, exhibiting similar distribution patterns. Furthermore, the LcMTA family, contained just two motifs and the distribution pattern of motifs began with motif 21 to 26 from N-terminal to C-terminal (Figure 2C). 

### 3.4. The miRNA Target Site Prediction

**Figure 3:**
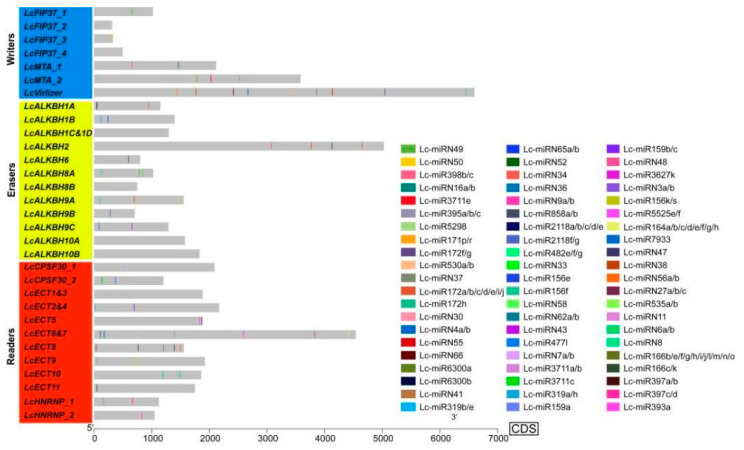
MiRNA target sites located on the CDS sequences of litchi m6A regulatory genes.

MiRNA target site prediction showed that a total of 77 miRNA target sites identified in 25 (80.6%) litchi m6A regulatory genes, and there were 19, 23, and 35 miRNA target sites located in the litchi m6A writers, erasers, and readers separately (Figure 3 and Table S3). Among all three kinds of litchi m6A regulatory genes, *LcVirlizer* in writers, *LcALKBH2* and *LcALKBH8A* in erasers, and *LcECT6&7* in readers occupied the largest number of miRNA targets, which could be targeted by 12, 5, 5, and 9 miRNAs separately. In the same kind of litchi m6A regulatory genes, some members that could be targeted by the same miRNA were identified. For example, *LcALKBH2* and *LcALKBH9A*, which belonged to the erasers, could be targeted by Lc-miR172a/b/c/d/e/i/j concurrently. *LcECT5* and *LcECT6&7*, which belonged to the readers, could be targeted by Lc-miRN48 simultaneously. The same miRNA could also be found to target genes that belonged to a different kind of litchi m6A regulatory gene, such as Lc-miR858a/b, which targeted the *LcECT6&7* and *LcALKBH6* concurrently. *LcFIP37_3*, *LcALKBH6*, *LcALKBH9B*, and *LcCPSF30_1* existed in just one miRNA target site. More generally, genes in either the same or different kinds of litchi m6A regulatory genes were targeted by different miRNAs. What interested us is that miRNA target sites located in the upstream sequence of *LcVirlizer*, *LcECT6&7*, and *LcECT8* functioned as ‘mRNA cleavage’ events, distributed in the downstream sequence, and acted as ‘translation repression’ events (Figure 3 and Table S3). 

### 3.5. Chromosomal Arrangement and GO Enrichment Analysis

**Figure 4:**
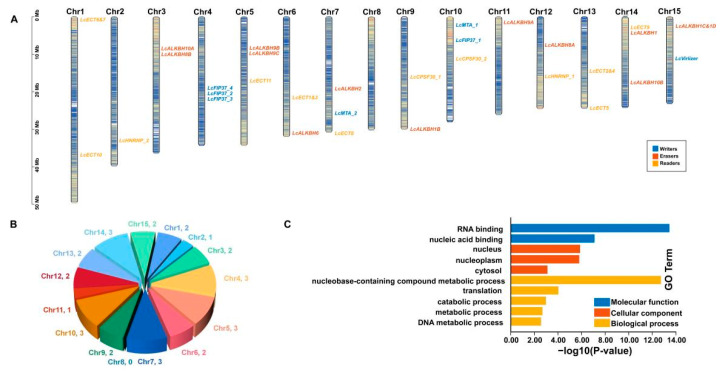
Chromosomal location and GO enrichment analysis of litchi m6A regulatory genes. (**A**) Chromosomal distribution.(**B**) Statistics of the number of litchi m6A regulatory genes on chromosomes. (**C**) GO enrichment analysis.

Chromosomal location analysis showed that litchi m6A writers, erasers, and readers were unevenly distributed on 15 different chromosomes, and the number of litchi m6A regulatory genes varied from 1 to 3 (Figure 4A). Statistical analysis found that Chr4, 5, 7, 10, and 14 embodied three genes; Chr1, 3, 6, 9, 12, 13, and 15 contained two genes; Chr2 and 11 only had one gene separately; and there was no gene identified on Chr8 (Figure 4A,B). GO enrichment analysis revealed that 31 litchi m6A regulatory genes functioned in molecular function, cellular component, and biological process (Figure 4C and Table S4). It was easily found that the m6A regulatory genes mainly functioned as nucleic acid binding (molecular function) in the nucleus (cellular component) and metabolic process (biological process). 

### 3.6. Cis-Regulatory Elements Analysis

**Figure 5:**
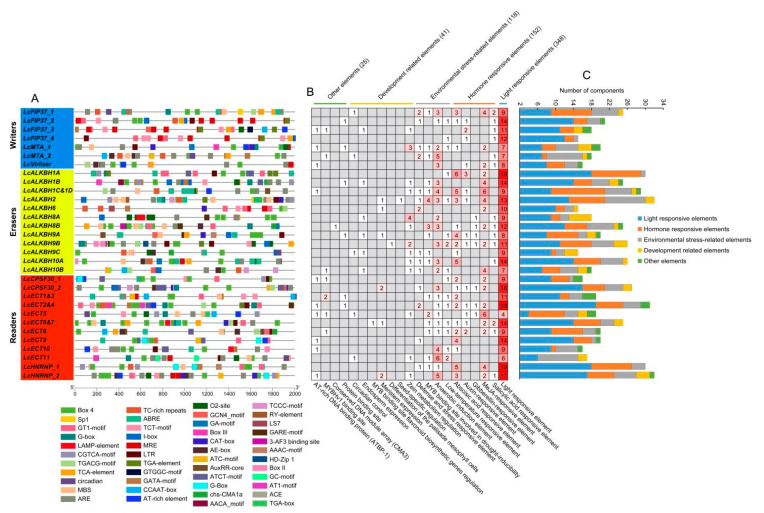
*Cis*-regulatory element analysis of litchi m6A regulatory genes. (**A**) The distribution of *cis*-regulatory elements. (**B**,**C**) show the statistics of *cis*-regulatory elements of each litchi m6A regulatory gene.

*Cis*-regulatory elements analysis exhibited that, with the exception of common elements, such as TATA-box, CAAT-box, TATC-box, and some unknown functional elements, a total of 684 *cis*-elements were identified in litchi m6A regulatory genes (Figure 5 and Table S5). Among these elements, the largest group was light responsive related, including 348 (50.88%) elements, such as GATA-motif, G-Box, GT1-motif, Box 4, TCT-motif, and MRE (MYB transcription factor binding site) elements. The second largest group was plant hormones related, comprising 152 (22.22%) elements, such as auxin responsive element (TGA-element and AuxRR-core), abscisic acid (ABA) response elements (ABRE), gibberellin (GA) response elements (GARE-motif), methyl jasmonate (MeJA) response elements (TGACG-motif and CGTCA-motif), and salicylic acid (SA) response elements (TCA-element). Among the plant-hormones-related elements, ABA response elements and MeJA-responsive elements were the largest two groups. The third largest group was about environmental stress related, embodying 118 (17.25%) elements, such as anaerobic induction element (ARE), anoxic specific inducibility (GC-motif), MYB binding site involved in drought inducibility (MBS), and low temperature responsive elements (LTR) (TC rich repeats). The fourth largest group was plant development related, possessing 41 (5.99%) elements, such as zein metabolism regulation (O_2_-site and CAT-box), circadian control (circadian), endosperm expression (GCN4_motif), differentiation of the palisade mesophyll cells (HD-Zip 1), meristem expression (CAT-box), MYB binding site involved in flavonoid biosynthesis (MBSI), and seed-specific regulatory element (RY-element). The smallest group was defined as other *cis*-elements, possessing 25 (3.65%) elements, such as AT-rich DNA-binding protein (AT-rich element), MYBHv1 binding site (CCAAT-box), conserved DNA module array (3-AF3 binding site), and protein-binding site (HD-Zip 3 and Box III).

### 3.7. The 3D Protein Structure Analysis

**Figure 6:**
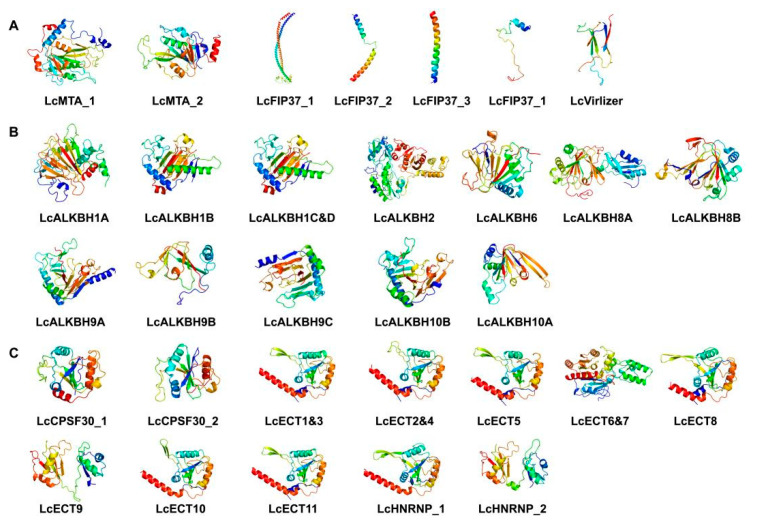
Prediction of three-dimensional domain of litchi m6A regulatory proteins (blue, green, yellow, orange, red, N-terminal to C-terminal). (**A**): Writers. (**B**): Erasers. (**C**): Readers.

Secondary structures analysis showed that litchi m6A regulatory proteins consisted of α-helix, extended chain (Ee), and random coil (Cc) (Table S6). For writers, α-helix occupied the largest proportion (22.34~77.88%), followed by random coiled amino acids (14.42%~66.03%) and extended chain (4.40%~16.29%). For erasers, random coiled amino acids occupied the largest proportion (45.82~74.15%), followed by α-helix (3.81%~37.53%) and extended chain (14.45%~22.03%). The readers exhibited a similar proportion ordering as the erasers; the largest proportion is random coiled amino acids (52.49%~68.29%), followed by α-helix (10.57%~31.34%) and extended chain (16.07%~28.00%). The 3D structures prediction revealed that the structures of Lc_ALKBH1B and Lc_ALKBH1C&D; the structures of Lc_ECT1&3, Lc_ECT2&4, Lc_ECT5, Lc_ECT8, Lc_ECT10, and Lc_HNRNP_1; and the structures of Lc_ECT8 and Lc_HNRNP_2 were similar, respectively, indicating that they shared functionality (Figure 6 and Table S7).

### 3.8. Expression Analysis of Litchi m6A Regulatory Genes by RNA-Seq Data

**Figure 7:**
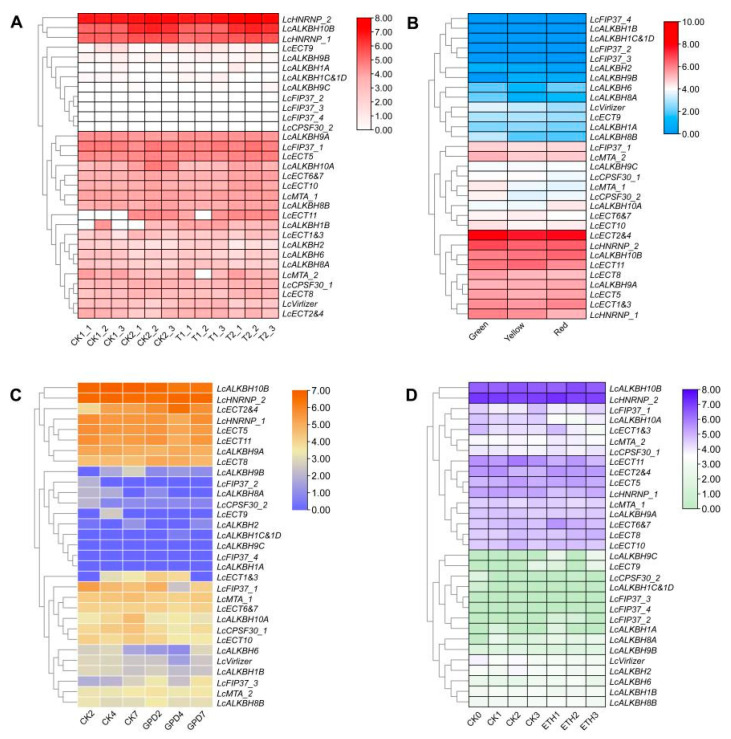
The expression pattern analysis of litchi m6A regulatory genes by RNA-seq data. (**A**) The peel coloring inhibition experiment of ‘Feizixiao’ variety treated by exogenous CPPU, CK: control group, T: CPPU treatment group. CK1 and T1: Green stage (35 d after anthesis), CK2 and T2: The best edible stage of fruit (67 d after anthesis). (**B**) Three different development stages of fruit of ‘Nuomici’ variety. Green: the peel is completely green; Yellow: yellow peel; Red: red peel. (**C**) The fruitlet samples of ‘Wuye’ variety after 2, 4, and 7 days, treated by girdling plus defoliation (GPD). (**D**) The abscission zone samples of ‘Feizixiao’ variety after 0, 1, 2, and 3 days, treated by exogenous ethephon (ETH).

According to the RNA-seq data described above (Table 1), the expression of litchi m6A regulatory genes was analyzed to investigate their potential function. The result showed a complicated expression pattern of litchi m6A regulatory genes during fruit maturation and abscission of litchi (Figure 7A–D). In order to obtain the key genes, differential expression analysis was also conducted as follows (Tables S8).

During the peel coloration inhibition experiment induced by exogenous CPPU, the expression of litchi m6A regulatory genes in the peel samples of the following two stages (complete green stage and the best edible stage) were inspected (Figure 7A and Table S8). Differential expression analysis showed that the expression of *LcMTA_2*, *LcALKBH1C&1D*, and *LcECT2&4* declined 1.22, 3.36, and 1.16 times significantly separately, while the expression of *LcALKBH10A* and *LcECT11* increased 1.04 and 9.36 times in the control group markedly, respectively. However, no genes exhibited significantly differential expression in the treatment group (Table S8).

During the green, yellow, and red stages of peel samples in the ‘Nuomici’ variety fruit (Figure 7B and Table S9), differential expression analysis found that the expression of *LcECT1&3* declined 24.61 and 18.71 times significantly in the yellow and red stage simultaneously, respectively. *LcECT9* and *LcALKBH10A* evidently increased 3.80 and 1.04 times in the yellow and red stage, respectively (Table S9).

During the period of fruitlet abscission of the ‘Wuye’ variety induced by GPD treatment, the expression of litchi m6A regulatory genes on 2, 4, and 7 days in the treatment groups, compared to the same time points of the control groups, exhibited as follows (Figure 7C and Table S10). Differential expression analysis revealed that the expression of *LcECT1&3* and *LcECT2&4* increased significantly (6.45 and 1.01 times) and (1.86 and 1.38 times) on the 2nd and 4th days, respectively, but *LcECT1&3* decreased 4.74 times on the 7th day significantly. The expression of *LcECT9*, *LcFIP37_1*, and *LcVirlizer* decreased 3.75, 2.37, and 1.67 times on the 4th day significantly, respectively. The expression of *LcALKBH10A* decreased 1.36 times on the 7th day significantly (Table S10).

During the period of fruitlet abscission of ‘Feizixiao’ variety induced by exogenous ETH treatment, the expression pattern of litchi m6A regulatory genes of abscission samples at 1, 2, and 3 days in the treatment groups, compared to the same time points in the control groups, exhibited as follows (Figure 7D and Table S11). Differential expression analysis showed that the expression of *LcALKBH10A* decreased 1.16 and 1.32 times significantly at 1 and 2 days after treatment, respectively. The expression of *LcALKBH9C* exhibited increased 4.72 and 5.06 times markedly at 1 and 3 days after treatment separately. The expression of *LcECT9* increased 4.21 times significantly at 1 day after treatment. The expression of *LcECT1&3* decreased 1.21 times significantly at 3 days after treatment (Table S11).

### 3.9. Expression Analysis of Litchi m6A Regulatory Genes by Quantitative qRT-PCR

**Figure 8:**
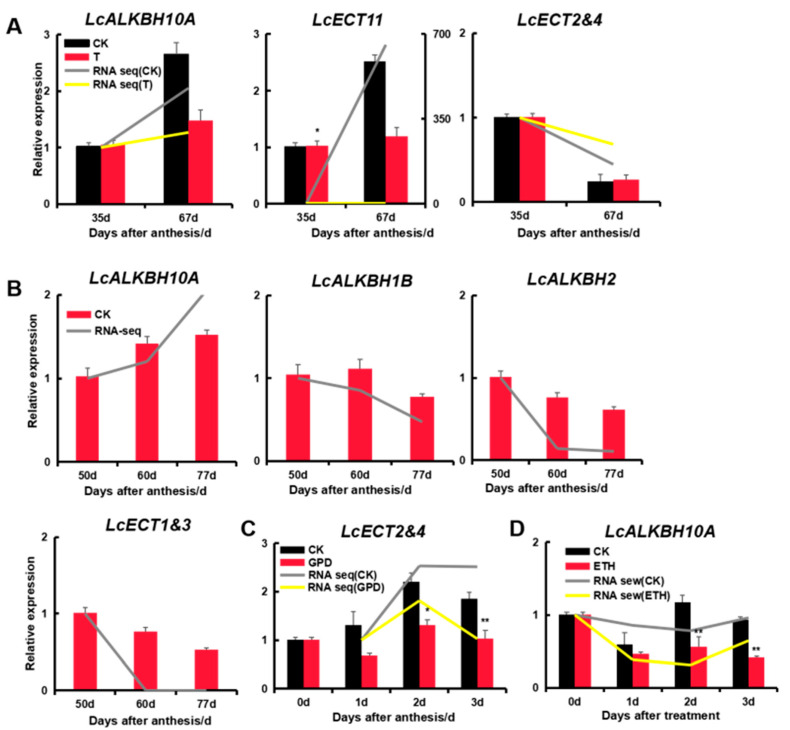
The expression pattern analysis of litchi m6A regulatory genes by qRT-PCR combining with RNA-seq data. (**A**) The samples corresponding to Figure 7A. (**B**) The expression of litchi m6A regulatory genes during three different development stages of ‘Nuomici’ variety fruit. Green: the peel is completely green (35 d after anthesis); Yellow: yellow peel (60 d after anthesis); Red: red peel (77 d after anthesis). (**C**) The expression of litchi m6A regulatory genes of fruit samples of ‘Feizixiao’ variety after 0, 1, 2, and 3 days, treated by girdling plus defoliation (GPD). (**D**) The expression of litchi m6A regulatory genes of abscission zone samples of ‘Feizixiao’ variety after 0, 1, 2, and 3 days, treated by exogenous ethephon (ETH). *: significant difference (*p* < 0.05); **: extremely significant (*p* < 0.01).

In order to further confirm the expression patterns of litchi m6A regulatory genes, the samples corresponding to the above four sets of RNA-seq data were re-collected in the orchard, and seven genes that showed significant difference in the RNA-seq data were selected randomly to evaluate by quantitative qRT-PCR. The results showed that the expression trend of these seven selected genes between qRT-PCR and RNA-seq were consistent with each other (Figure 7 and Figure 8 and Tables S9–S12), proving that the outcomes we obtained were reliable.

## 4. Discussion

In this study, a total of 31 litchi m6A regulatory genes were ultimately authenticated from the litchi genome that has been published recently [21], including 7 m6A writers, 12 m6A erasers, and 12 readers (Table 2). Compared with other plants, the number of litchi m6A regulatory genes was similar to arabidopsis (32) [29] and tea (Camellia sinensis) (34) [30], indicating that the reliability of our results was adequate. Phylogenetic relationships analysis showed that all of the litchi m6A writers, erasers, and readers could be divided into three groups separately (Figure 1). However, some members or entire groups were lost, and other groups obtained new members in the litchi genome (Table 2 and Figure 1). For example, the MT family of the litchi m6A writers only obtained the LcMTA subfamily but lost the LcMTB and LcMTC subfamilies in litchi (Table 2 and Figure 1A), which was consistent with the identification result of the pteridophyte [30]. When it comes to the other groups of writers, there were four LcFIP37s (LcFIP37_1-4) of the FIP37 subfamily (group 2) and one LcVirlizer (group 3) of the VIR subfamily obtained in litchi, but just one FIP37 was identified and an entire VIR subfamily was lost in pteridophyte [30]. A similar phenomenon could also be found in the litchi m6A erasers and readers as well. These results described above suggested that litchi m6A regulatory genes had experienced gene expansion and loss events during their evolutionary history. Thus, we could speculate that new m6A genes acquired in litchi might be used to enhance the benefits of m6A modification, and the gene loss might be due to their biological functions that could be taken over by other m6A genes during their adaption to the changes induced by the external environment or while exiting other alternative mechanisms for m6A methylation; however, more data is needed to support this. Conserved domain analysis had confirmed that the typical domains belonged to each group of the m6A regulatory genes that could be found in the corresponding litchi m6A regulatory genes (Figure 2B and Table S2). The distribution of domains and motifs exhibited similar patterns in the same group (Figure 2B,C, Tables S2 and S3), suggesting that these genes might share similar function. 

The m6A modification had been considered to play an important role in the post-transcriptional regulation of gene expression in the eukaryotes [1]. MiRNA, of about 19–24 nt, a kind of small noncoding RNA, acts as a post transcriptional regulator and plays a critical role during the development of plant [31]. Recent research revealed that m6A modification could function during the miRNA biosynthesis processing in multiple species [32]. In our result, 25 (80.6%) litchi m6A regulatory genes pertained to 77 miRNA target sites, indicating that the m6A modification could also be regulated by miRNA at the post-transcriptional level. Furthermore, miRNA target sites located in the upstream sequence (functioning as ‘mRNA cleavage’ events) and downstream sequence (functioning as ‘translation repression’ events) of *LcVirlizer*, *LcECT6&7*, and *LcECT8* (Figure 3 and Table S3) probably form the two ‘hits’ way for triggering phasiRNA biogenesis, as described previously [33]. GO annotation and enrichment analysis revealed that the function of 31 litchi m6A regulatory genes mainly focused on nucleic acid binding (molecular function), nucleus (cellular component), and metabolic process (biological process) (Figure 4C and Table S4). Gene expression could be regulated by cis-regulatory elements on transcriptional level [34]. The statistical results of cis-regulatory elements unfolded that the transcription initiation of litchi m6A regulatory genes might be mainly in response to light, plant hormones (auxin, ABA, GA, MeJA, and SA), environmental stress, and plant development (Figure 5 and Table S5). In order to unearth the function of litchi, m6A regulatory genes during fruit development of litchi, RNA seq data involving in peel coloration, and fruit abscission were adopted to perform further analysis. 

It is well known that the peel coloration is one of the most vital factors to declaring the maturation degree and determining the market acceptance of large of fruits. Fruit coloration of litchi is mainly determined by the accumulation of anthocyanins, and the degradation of carotenoids and chlorophyll are induced by a large of structure genes and transcription factors related to the biosynthesis and degradation of the above three pigments [24,35,36,37]. Recent research showed that m6A modification levels would gradually decrease during the natural ripening stage of tomato fruits, but this ripening process could be accelerated by reducing the m6A modification levels through exogenous DA (m6A writer inhibitor) treatment and delayed by increasing the m6A modification levels induced by exogenous (m6A eraser inhibitor) treatment [12,13]. In this study, during the experiment of the fruit coloration inhibition of the ‘Feizixiao’ variety induced by exogenous CPPU, the writer (*LcMTA_2*) declined significantly, while the eraser (*LcALKBH10A*) increased markedly in the control group. However, no genes were significantly expressed in the treatment group (Figure 7A and Table S8). This suggested that higher m6A modification levels induced by a relatively higher expression of *LcMTA_2* and lower expression of *LcALKBH10A* might be the important factors for the delay of the maturation of the ‘Feizixiao’ variety induced by exogenous CPPU. CPPU is an inhibitor of ABA biosynthesis, and it had been reported that the application of exogenous CPPU had no significant effect on fruit weight, total sugar, and organic acids, but it did repress the coloration conspicuously by suppressing chlorophyll loss and anthocyanin accumulation in the fruit of the ‘Feizixiao’ variety [38]. ABA was considered as the critical hormone related to anthocyanin accumulation and chlorophyll loss during litchi fruit ripening [19,24,36,39], and the fruit of the ‘Feizixiao’ variety that appeared in the ‘stay green’ phenomenon (an uneven pigmenting problem) was considered to be mainly caused by the insufficient anthocyanin content induced by the relative low ABA accumulation during fruit coloration, which result in could not cover up the chlorophyll related determining substances of background color [35]. In addition, a study published lately demonstrated that m6A modification regulated the fruit ripening process of strawberry in an abscisic acid (ABA)-dependent manner [40], This implied a similar regulatory mechanism to that in litchi, but it needs further work to confirm this suspicion. Compared to the ‘Feizixiao’ variety, the ‘Nuomici’ variety could fulfil coloration. During the green-to-red stage of the ‘Nuomici’ variety fruit, no significant difference was found in the writers, but an eraser (*LcALKBH10A*) increased evidently in the yellow and red stage, and the TPM and count value in the red stage were much larger in the same stage of the ‘Feizixiao’ variety (Figure 7B and Table S9). This result suggested that the eraser (*LcALKBH10A*) is a key factor to determining the difference of coloration between the two varieties, but it also needs further research to ascertain whether this is the case. Fruit drop is one of the major factors for deciding the ultimate yield in litchi industry. It is common knowledge that there are 3–5 physiological fruit drop waves (I, II, III, IV, and V) in different litchi cultivars, and these process were mainly induced by poor pollination and fertilization, carbohydrate stress, high ABA and ETH level, low indole-3-acetic acid (IAA) content, low cytokinins (CTKs) and gibberellins (GAs), and so on [20]. Zhao et al. (2020) systematically summarized the current molecular mechanism of the litchi fruit drop. There is sufficient evidence to demonstrate that the regulation of gene expression by m6A principally accords to the regulation of RNA processing, splicing, nucleation, translation, and stability [1]. However there is no direct evidence of m6A methylation modification and plant abscission nowadays. Combined with large amounts of light, plant hormones, environmental stress, and plant development related cis-regulatory elements located in the promoter region of litchi m6A regulatory genes, there is a remarkable reaction of some litchi m6A regulatory genes to the fruit abscission induced by GPD and exogenous ETH treatment. We predict that m6A modification might play a fundamental role during the abscission of litchi, but it still need more works to be confirmed. 

## 5. Conclusions

In conclusion, 31 litchi m6A regulatory genes (including 7 m6A writers, 12 m6A erases, and 12 readers) were identified with the help of a bioinformatics tools and the litchi genome primarily. Additionally, their physicochemical properties, including their phylogenetic relationship, gene and protein structure, miRNA target site, chromosomal arrangement, and *cis*-regulatory elements, were analyzed as part of a comprehensive survey. Expression analysis revealed that litchi m6A regulatory genes might be related to the peel coloration and fruit abscission of litchi. Our results will supply some insight into the characteristics of litchi m6A regulatory genes and their probable epigenetic regulation mechanism in litchi.

## Figures and Tables

**Table 1:** Plant materials used for expression analysis.

Plant Materials for RNA-Seq Analysis								
Data Set	Variety	Sample ID	Samples	Biological Replicates	Platform	LibraryLayout	Accesion Number	Data Sources
1	‘Feizixiao’ (27-year-old)	CK1_1, CK1_2, CK1_3, CK2_1, CK2_2,CK2_3, T1_1, T1_2, T1_3, T2_1, T2_1, T2_1.	Peel: CK1 and T1: Green stage (35 d after anthesis); CK2 and T2: (57 d after anthesis).	3	ILLUMINA	PAIRED		Home data
2	‘Nuomici’ (Adult tree)	Green (SRX700596), Yellow (SRX700598), Red (SRX700599).	Peel: Green: 52 d after anthesis; Yellow: 62 d after anthesis; Red: 72 d after anthesis. Grown in normal conditions.	1 (mixed sample of 3 biological replicates)	ILLUMINA	SINGLE	SRP047115	Lai et al., 2015 [24]
3	‘Wuye’ (9-year-old)	CK2 (SRX847812), CK4 (SRX847822), CK7(SRX847823), GPD2 (SRX847824), GPD4 (SRX847825), GPD7(SRX847826).	Fruitlet: CK: Control group; GPD: Treatment group; 2, 4, and 7 d represented fruitlet abscission at 2, 4, and 7 days after being treated with GPD treatment at 35 days after anthesis.	1 (Mix sample)	ILLUMINA	SINGLE	SRA234477	Li et al., 2015 [25]
4	‘Feizixiao’ (9-year-old)	CK0 (SRX5126892), CK1 (SRX5126893), CK2 (SRX5126894), CK3 (SRX5126895), ETH1 (SRX5126896), ETH2 (SRX5126897), ETH3 (SRX5126898)..	Abscission zone: CK: Control group; GPD: Treatment group; 0, 1, 2, and 3 d represented fruitlet abscission at 0, 1, 2, and 3 days after being treated with 250 mg/L ethephon solution at 25 days after anthesis.	1	ILLUMINA	SINGLE	SRP173341	Li et al., 2015 [26]
**Plant materials for qRT-PCR analysis**								
1	‘Feizixiao’ (27-year-old)	35 d and 67 d.	Peel collected at 35 and 67 days treated with 4 mg/L CPPU after anthesis.	3				Home data
2	‘Nuomici’ (27-year-old)	50 d, 60 d, and 77 d.	Peel collected at 50, 60, and 77 days after anthesis, which grew in normal condition.	3				Home data
3	‘Feizixiao’ (27-year-old)	0 d, 1 d, 2 d, and 3 d.	Fruitlet collected at 0, 1, 2, and 3 days treated with GPD treatment at 35 days after anthesis.	3				Home data
4	‘Feizixiao’ (27-year-old)	0 d, 1 d, 2 d, and 3 d.	Abscission zone tissues collected at 0, 1, 2, and 3 days treated with 250 mg/Lexogenous ethephon (ETH) after anthesis.	3				Home data

**Table 2:** Basic information of litchi m6A writers, erasers, and readers.

Genes	Gene ID in Genome	Number of Amino Acids (aa)	PI	MW (kDa)	Instability Index	Aliphatic Index	Grand Average of Hydropathicity (GRAVY)	Subcellular Localization Prediction
**Writers**								
*Lc_FIP37_1*	*LITCHI022446.m2*	341	5.13	38.74	56.79	79.88	−0.83	Nucleus
*Lc_FIP37_2*	*LITCHI031231.m1*	104	7.86	11.82	50.26	93.94	−0.591	Nucleus
*Lc_FIP37_3*	*LITCHI031301.m1*	109	4.9	12.39	43.18	95.78	−0.299	Nucleus
*Lc_FIP37_4*	*LITCHI031225.m1*	165	5.83	18.67	47.28	99.33	−0.358	Nucleus
*Lc_MTA_1*	*LITCHI021922.m2*	706	5.71	78.63	47.91	76.4	−0.526	Nucleus
*Lc_MTA_2*	*LITCHI009397.m2*	1195	7.68	134.28	60.59	40.72	−1.349	Nucleus
*Lc_Virlizer*	*LITCHI018982.m2*	2200	5.41	240.57	52.3	96.91	−0.079	Nucleus
**Erasers**								
*Lc_ALKBH1A*	*LITCHI004660.m2*	384	8.05	43.52	61.01	79.3	−0.287	Cytoplasm
*Lc_ALKBH1B*	*LITCHI029924.m2*	466	7.72	51.25	45.99	66.31	−0.72	Nucleus
*Lc_ALKBH1C&1D*	*LITCHI018059.m1*	432	9.51	48.40	53.24	77.36	−0.516	Nucleus
*Lc_ALKBH2*	*LITCHI008899.m3*	1676	8.86	190.29	42.36	82.82	−0.362	Plasma membrane
*Lc_ALKBH6*	*LITCHI004157.m1*	267	6.25	29.92	47.8	89.36	−0.281	Nucleus
*Lc_ALKBH8A*	*LITCHI020056.m1*	342	6.51	38.28	51.41	80.91	−0.256	Chloroplast
*Lc_ALKBH8B*	*LITCHI026423.m1*	250	4.75	28.35	52.21	78	−0.406	Nucleus
*Lc_ALKBH9A*	*LITCHI006480.m1*	519	5.76	57.89	54.08	73.04	−0.638	Nucleus
*Lc_ALKBH9B*	*LITCHI000266.m1*	236	8.85	26.13	66.51	81.74	−0.242	Cytoplasm
*Lc_ALKBH9C*	*LITCHI000270.m1*	430	8.38	48.34	55.71	81.33	−0.443	Nucleus
*Lc_ALKBH10A*	*LITCHI026300.m1*	526	5.64	58.08	54.42	80.82	−0.421	Chloroplast
*Lc_ALKBH10B*	*LITCHI005944.m1*	610	8.25	66.15	58.06	70.97	−0.541	Nucleus
**Readers**								
*Lc_ECT1&3*	*LITCHI003339.m2*	628	6.23	68.53	37.06	57.75	−0.731	Nucleus
*Lc_ECT2&4*	*LITCHI024309.m2*	724	6.96	79.28	33.38	57.64	−0.714	Nucleus
*Lc_ECT5*	*LITCHI025245.m1*	629	5.33	68.72	48.82	54.74	−0.716	Nucleus
*Lc_ECT6&7*	*LITCHI014289.m1*	1514	5.63	168.82	4.02	75.09	−0.44	Chloroplast
*Lc_ECT8*	*LITCHI009804.m1*	520	6.83	57.50	36.75	58.56	−0.583	Nucleus
*Lc_ECT9*	*LITCHI004655.m12*	641	6.83	72.08	47.45	70.23	−0.5	Extracellular space
*Lc_ECT10*	*LITCHI016506.m1*	620	5.28	68.19	44.72	63.39	−0.694	Nucleus
*Lc_ECT11*	*LITCHI000687.m1*	584	6.62	65.00	28.17	52.43	−0.864	Nucleus
*Lc_HNRNP_1*	*LITCHI020595.m1*	375	6.27	38.96	25.54	39.81	−0.74	Nucleus
*Lc_HNRNP_2*	*LITCHI013653.m1*	350	9.07	36.25	30.65	44.57	−0.678	Nucleus
*Lc_CPSF30_1*	*LITCHI028685.m1*	697	6.12	75.97	54.57	42.31	−0.859	Nucleus
*Lc_CPSF30_2*	*LITCHI022917.m5*	402	5.85	45.82	57.48	64.78	−0.807	Nucleus

## Data Availability

Data are contained in the article or supplementary files.

## Supplementary Materials

The following supporting information can be downloaded at: https://www.mdpi.com/article/10.3390/genes13122284/s1, Table S1: Primers of selected litchi m6A writers, erasers and readers genes and reference genes; Table S2: Position information of domain and motif of litchi m6A writers, erasers and readers genes; Table S3: The potential miRNA target sites of litchi m6A writers, erasers and readers genes; Table S4: GO enrichment analysis of litchi m6A writers, erasers and readers genes; Table S5: *Cis*-acting element information in the promoter region of litchi m6A writers, erasers and readers genes; Table S6. Two-dimensional structures of litchi m6A writers, erasers and readers genes; Table S7: Three-dimensional structure annotations of litchi m6A writers, erasers and readers proteins; Table S8: The, count and CPM value, differential expression analysis of litchi m6A writers, erasers and readers genes during peel coloring induced by exogenous CPPU in ‘Feizixiao’ variety; Table S9: The, count and CPM value, differential expression analysis of litchi m6A writers, erasers and readers genes during three different development stages of fruit in ‘Nuomici’ variety; Table S10: The, count and CPM value, differential expression analysis of litchi m6A writers, erasers and readers genes after 2, 4 and 7 days treated by girdling plus defoliation of fruit samples in ‘Wuye’ variety; Table S11: The count, and CPM value, and differential expression analysis of litchi m6A writers, erasers and readers genes after 0, 1, 2, and 3 days treated by exogenous ethephon of abscission zone samples in ‘Feizixiao’ variety.
